# Surrogate biomarkers of outcome for wake-up ischemic stroke

**DOI:** 10.1186/s12883-022-02740-z

**Published:** 2022-06-09

**Authors:** Pablo Hervella, María Luz Alonso-Alonso, María Pérez-Mato, Manuel Rodríguez-Yáñez, Susana Arias-Rivas, Iria López-Dequidt, José M. Pumar, Tomás Sobrino, Francisco Campos, José Castillo, Ramón Iglesias-Rey

**Affiliations:** 1 Neuroimaging and Biotechnology Laboratory (NOBEL), Clinical Neurosciences Research Laboratory (LINC), Health Research Institute of Santiago de Compostela (IDIS), Santiago de Compostela, Spain; 2 Hospital Clínico Universitario, Rúa Travesa da Choupana, s/n, 15706 Santiago de Compostela, Spain; 3 Neuroscience and Cerebrovascular Research Laboratory, Department of Neurology and Stroke Center, La Paz University Hospital, Neuroscience Area of IdiPAZ Health Research Institute, Universidad Autónoma de Madrid, Madrid, Spain; 4 Stroke Unit, Department of Neurology, Hospital Clínico Universitario, Santiago de Compostela, Spain; 5 Department of Neuroradiology, Hospital Clínico Universitario, Health Research Institute of Santiago de Compostela (IDIS), Santiago de Compostela, Spain; 6 Neuroaging Laboratory (NEURAL), Clinical Neurosciences Research Laboratory (LINC), Health Research Institute of Santiago de Compostela (IDIS), Santiago de Compostela, Spain; 7 Translational Stroke Laboratory (TREAT), Clinical Neurosciences Research Laboratory (LINC), Health Research Institute of Santiago de Compostela (IDIS), Santiago de Compostela, Spain

**Keywords:** Biomarker, Stroke prevention, Prognosis, Vitamin D, Wake-up stroke

## Abstract

**Background:**

Wake-up ischemic stroke (IS) has been usually excluded from acute stroke therapy options for being outside of the safe treatment window. We identified risk factors, and clinical or molecular biomarkers that could be therapeutic targets for wake-up stroke prevention, thus hopefully leading to a decrease in its mortality and disability in medium to long-term outcome.

**Methods:**

4251 ischemic stroke (IS) patients from a prospectively registered database were recruited; 3838 (90.3%) had known onset-symptom time, and 413 (9.7%) were wake-up strokes. The main endpoint was to analyze the association between different serum biomarkers with wake-up IS episodes and their progression. Leukocytes count, serum levels of C-reactive protein, fibrinogen, interleukin 6 (IL-6), and vitamin D were analyzed as inflammation biomarkers; N-terminal pro-B-type Natriuretic-Peptide and microalbuminuria, used as atrial/endothelial dysfunction biomarkers; finally, glutamate levels as excitotoxicity biomarker. In addition, demographic, clinical and neuroimaging variables associated with the time-evolution of wake-up IS patients and functional outcome at 3 months were evaluated. Good and poor functional outcome were defined as mRS ≤2 and mRS > 2 at 3 months, respectively.

**Results:**

Wake-up IS showed a poorer outcome at 3-months than in patients with known on-set-symptom time (59.1% vs. 48.1%; *p* < 0.0001). Patients with wake-up IS had higher levels of inflammation biomarkers; IL-6 levels at admission (51.5 ± 15.1 vs. 27.8 ± 18.6 pg/ml; *p* < 0.0001), and low vitamin D levels at 24 h (5.6 ± 5.8 vs. 19.2 ± 9.4 ng/ml; *p* < 0.0001) are worthy of attention. In a logistic regression model adjusted for vitamin D, OR was 15.1; CI 95%: 8.6–26.3, *p* < 0.0001. However, we found no difference in vitamin D levels between patients with or without clinical-DWI mismatch (no: 18.95 ± 9.66; yes: 17.84 ± 11.77 ng/mL, *p* = 0.394). No difference in DWI volume at admission was found (49.3 ± 96.9 ml in wake-up IS patients vs. 51.7 ± 98.2 ml in awake IS patients; *p* = 0.895).

**Conclusions:**

Inflammatory biomarkers are the main factors that are strongly associated with wake-up IS episodes. Wake-up IS is associated with lower vitamin D levels. These data indicate that vitamin D deficiency could become a therapeutic target to reduce wake-up IS events.

## Background

Between 8 and 39% of strokes are wake-up strokes i.e., they occur during sleep [1–3]. The concept of wake-up stroke emerged because of the limitations of the intravenous recombinant tissue plasminogen activator (rtPA) treatment (time window < 4.5) [2–5]. This means that the time when patients were last known well is used as a reference time for the stroke onset. Consequently, wake-up ischemic stroke (IS) has been usually excluded from acute stroke therapy options for being outside of the safe treatment window. However, different comparisons between wake-up and awake ischemic strokes have demonstrated clinical and radiological similarities between them [5–7]. In this line, studies on the timing of the onset-symptoms of ischemic stroke determined that there is a peak incidence in the early morning hours, suggesting that many wake-up strokes probably occur close to awakening [8, 9]. Proposed physiologic mechanisms related to this type of stroke include sleep-disordered breathing, patent foramen ovale, new-onset atrial fibrillation, endothelial dysfunction, morning blood pressure surge, peak in pro-thrombotic factors and increased platelet aggregation [10–12]. Analyzing the possible risk factors related to these events, as well as the methods to determine stroke symptom onset time by using imaging techniques or biomarkers, is a clinical priority. As a result, different research works and clinical trials have recently been developed, performed in imaging-selected patients, which reported better functional outcome in terms of mRS and NIHSS in wake-up ischemic individuals [13–16].

In recent years, advanced neuroimaging techniques have led to the development of a variety of research works and clinical trials seeking to include many of these patients with undetermined stroke onset in reperfusion therapies. To replace the old onset time window concept, tissue time window was based on brain tissue post-ischemic changes under the guidance of neuroimaging, mainly through Magnetic Resonance Imaging (MRI) and Computed Tomography (CT) [17–19]. In this sense, WAKE-UP trial demonstrated that intravenous thrombolysis with alteplase resulted in a better functional outcome than treatment with placebo among acute ischemic stroke patients with unknown onset-symptom time based on MRI guide (DWI-FLAIR mismatch) [13, 14]. The EXTEND trial, based on CT perfusion or PWI-DWI MRI, found efficacy in rtPA treatment in patients with a favorable perfusion imaging profile 4.5 to 9 hours after stroke onset or on awakening [15]. Another single-center study suggested that thrombolytic therapy is effective in reducing infarct volume and improve functional outcome without increasing hemorrhagic transformation in patients with wake-up strokes selected by CT [16].

Other clinical aspects and factors that can help understand the mechanisms behind wake-up stroke and prevent its occurrence or its consequences are less known. In line, the cerebrovascular endothelium represents a biological and mechanical barrier between the cerebral and vascular compartments, where the endothelial dysfunction has been related to wake-up IS episodes [20, 21]. Most neurovascular risk factors, both traditional and newly identified, are linked to endothelial dysfunction in a cumulative manner, such as vitamin D deficiency. Vitamin D insufficiency has been associated to major chronic diseases such as cardiovascular and neurological disorders, diabetes, and cancer, all of which are linked to oxidative stress, inflammation, and aging [22]. It would be beneficial to identify clinical or biological markers related to the cardiovascular system (inflammation, excitotoxicity, endothelial and atrial dysfunction biomarkers), as well as risk factors that could become therapeutic targets for the prevention of wake-up stroke, resulting in lower mortality and disability in the medium to long term. We hypothesized that elevated serum levels of inflammation, excitotoxicity, endothelial and atrial dysfunction biomarkers might be involved in a higher frequency of wake-up IS episodes. In the present study, we looked at clinical factors to identify biomarkers associated to wake-up IS events, with a particular focus on vitamin D’s role.

## Methods

### Patient selection

For this study, the inclusion criteria were the following: IS patients confirmed by neuroimaging, attended by a neurologist according to national and international guidelines [23] and admitted to the Stroke Unit, and prospectively registered in an approved data bank. This data bank contains demographic, clinical, analytical and imaging variables for stroke patients treated in the Stroke Unit. This data bank has been pseudomised and communicated to the Hospital Management for its use for research purposes. Exclusion criteria were the following: 1) death during the first 24 hours; 2) transfer of the patient to other health care center; 3) lack of at least two neuroimaging studies in the first week; 4) known chronic inflammatory or infectious diseases (fever of unknown origin (axillary temperature > 37.5 °C), inflammatory or infectious disease, etc); and 5) loss of follow-up (personal interview or telephone) at 3 months. The analysis of the data for this study was retrospective (January 2008–December 2017). All patients or their relatives signed the informed consent for inclusion in the registry and for anonymous use.

### Study design

The main objective of this study was to analyze the association between inflammation, excitotoxicity, endothelial and atrial dysfunction biomarkers with wake-up IS episodes. As a secondary objective, we analyzed demographic, clinical and neuroimaging variables associated with the time-evolution of wake-up IS patients and functional outcome at 3 months.

### Demographic and clinical variables

We defined wake-up IS as an ischemic stroke that is associated with neurological symptoms on awakening. By definition, the patient’s last-know-well time corresponds to the onset of sleep on the evening before event [2]. The demographic variables evaluated were: age, history of high blood pressure (at least 2 blood pressure measurements > 140/85 mmHg or under antihypertensive treatment), diabetes (previous diagnosis or under anti-diabetic treatment), smoking (habitual smoker or up to the previous half year), alcohol consumption (> 350 g of alcohol/week), dyslipidemia (at least a previous measurement of total cholesterol > 230 mg/dL or lipid lowering therapy), coronary disease, peripheral arterial disease, atrial fibrillation, carotid stenosis, hemodynamic carotid stenosis, occlusion/carotid sub-occlusion, prior transient ischemic attack, and treatment with anticoagulants / antiplatelets.

As clinical variables we analyzed the National Institute of Health Stroke Scale (NIHSS) [24] at admission, at 24 h, at discharge and at 3 months, previous and at 3-months±15 days modified Rankin Scale (mRS) [25], time detection-emergencies (time between the moment of the identification of the symptoms (not the beginning of the stroke) and the attention in the emergency room by a neurologist of the Stroke Unit), axillary temperature at admission, reperfusion treatments (intraarterial or intravenous fibrinolysis, thrombectomy, and both processes), and time from detection to rtPA treatment. Good and poor functional outcome were defined as mRS score of ≤2 and mRS > 2 at 3 months, respectively. We defined early neurological improvement as a decrease ≥8 points in the NIHSS in the first 24 hours with respect to baseline NIHSS score. By contrast, early neurological deterioration was defined as an increase of four points in the first 48 hours (≥ 4 points). Both scales were evaluated by internationally certified neurologists and supervised by the same neurologist.

### Neuroimaging studies

Neuroimaging studied in IS included the baseline infarct volume (DWI-lesion) and the presence of clinical-DWI mismatch (defined as NIHSS ≥8 and DWI volume ≤ 25 ml) [26]. Lesion volumes were calculated by using ABC/2 method [27] until 2016 and through automated planimetric method afterwards. IS etiology was evaluated according to TOAST criteria [28]. Expert neuroradiologists blinded to clinical data performed neuroimaging evaluations.

### Molecular markers

Blood sample determinations that were carried out were as follows: erythrocyte sedimentation rate (ESR), glucose, triglycerides, glycated hemoglobin, LDL / HDL cholesterol. The inflammation markers included were fibrinogen, C-reactive protein, leukocytes, erythrocyte sedimentation rate, serum levels of interleukin 6 (IL-6) [29], vitamin D levels (25-Hydroxy-Vitamin D) at 24 hours [30]. N-terminal pro-B-type Natriuretic Peptide (NT-proBNP) and microalbuminuria were the atrial dysfunction and endothelial markers determined, respectively [31]. Finally, serum levels of glutamate were evaluated as excitotoxicity marker [32].

Coagulation tests, hematology, and biochemistry, were determined in the central laboratory of the Hospital blinded to clinical / neuroimaging data. Serum glutamate and IL-6 were performed in the Clinical Laboratory by researchers blinded to clinical / neuroimaging data. Blood samples, obtained from all patients at admission, were collected in test tubes, centrifuged at 3000 g during 15 min and immediately frozen / stored (at − 80 °C). Serum concentrations of IL-6 were determined by enzyme linked immunosorbent assay (ELISA) technique following manufacturer’s instructions (BioLegend, San Diego, USA), minimum assay sensitivity 1.6 pg/ml with an intra- and inter-assay coefficient of variation (5.0% vs. 6.8%). Serum glutamate concentration was determined by high performance liquid chromatography (1260 Infinity II, Agilent Technologies, Santa Clara, California, USA) using the AccQ-Tag™ Precolumn derivatization method for amino acid analysis (Waters, Milford, MA, USA), following a previously described method [33]. Biomarkers were evaluated within the first 3 months after blood sample collection and store. However only in 61.2% of patients serum glutamate was analyzed, IL-6 in 80.2% of patients and vitamin D in 79.8% of patients.

### Statistical analysis

Results were described as mean ± standard deviation or the median [range] according to the type of distribution obtained by the Kolmogorov-Smirnov test for a sample with Lilliefors (correction of significance). Qualitative variables (factors) were described as percentages (%). The significance of the differences was estimated using the student’s t-test / the Mann-Whitney U-test. For the differences we used the chi-square test and, if applicable, the uncertainty coefficient.

In order to evaluate the independent variables associated with wake-up IS a multiple regression models were used, identifying continuous / categorical variables. First, we performed logistic regression models including variables (factors) with significant differences (*p* < 0.05) in univariate studies grouped according to demographic, clinical and neuroimaging variables. With the selected factors, a new logistic regression model was developed, including the result of the biomarkers analyses. Bivariate correlations were performed using Spearman or Pearson’s coefficients. Based on the relationship between inflammation and vitamin D levels previously described [34, 35], two models for multivariate analyses with or without the value of IL-6 as an independent variable were performed. In the first model we evaluated if low vitamin D levels were more frequent in wake-up IS patients. The inclusion of IL-6 levels in the second model helped to confirm that this effect is dependent on increased inflammation.

To detect the capacity of vitamin D to categorize the values related with wake-up IS, Receiver Operating Characteristic (ROC) curves were evaluated, converting continuous variables into categorical ones for a value that offers maximum sensitivity / specificity. In turn, clinical-DWI mismatch and early neurological improvement relationship patients with IS according to vitamin D levels were evaluated taking into account a logistic regression model adjusted for age, previous mRS, temperature, C-reactive protein, NIHSS on admission and fibrinolytic treatment. Results were showed as odds ratio (OR) with 95% confident intervals (CI 95%). A *p* value < 0.05 was considered statistically significant. Data were analyzed using IBM SPSS_v.25 (IBM, Chicago, IL, USA) for Mac.

## Results

Four thousand seven hundred eighty-six patients were enrolled for the present study. We excluded 97 patients who died during the first 24 hours, 118 who were transferred to other health care center, 33 known chronic inflammatory or infectious diseases, and 287 with no follow-up at 3 months. The final recruited sample was therefore 4251 IS patients eligible for the analysis. Of these IS, 3838 (90.3%) had a known onset-symptom time and 413 (9.7%) were wake-up strokes (mean ages 72.0 ± 13.8 and 71.9 ± 15.7, respectively). The demographic, clinical, neuroimaging, molecular and outcome variables for patients with awake versus and wake-up IS patients are detailed in Table 1.

**Table 1:** Univariate analysis. Demographic, clinical, neuroimaging, molecular and outcome for patients with awake and wake-up IS (*n* = 4251)

	Awake IS (*n* = 3838)	Wake-up IS (*n* = 413)	*p* value
**Demographic variables**			
Age, years	72.0 ± 13.8	71.9 ± 15.7	0.003
Female gender, %	45.3	48.2	0.275
Arterial hypertension, %	62.7	64.2	0.592
Diabetes, %	24.2	26.4	0.335
Smoking, %	16.0	17.7	0.399
Alcohol consumption, %	11.5	12.1	0.686
Hyperlipidemia, %	33.6	39.5	0.019
Peripheral arterial disease, %	5.8	4.1	0.178
Ischemic heart disease, %	11.0	11.6	0.741
Atrial fibrillation, %	20.3	25.9	0.011
Heart failure, %	4.0	5.8	0.092
Previous carotid stenosis, %	1.5	0.5	0.119
Ipsilateral hemodynamic carotid Stenosis, %	17.0	20.7	0.510
Occlusion/carotid sub-occlusion, %	15.1	15.4	0.897
Previous transient ischemic attack, %	5.0	3.4	0.184
Previous antiaggregants, %	23.8	25.4	0.466
Previous anticoagulants, %	7.5	12.3	0.001
**Clinical, Neuroimaging features**			
mRS (Previous)	0 [0, 1]	0 [0, 2]	< 0.0001
NIHSS (at admission)	16 [12, 20]	18 [13, 20]	0.013
NIHSS (at 24 h)	7 [3, 13]	8 [4, 14]	0.003
NIHSS (at discharge)	7 [2, 12]	9 [3, 14]	< 0.0001
Time detection (awakening)-emergencies, minutes	237.4 ± 164.2	293.5 ± 112.8	0.137
Maximum axillary temperature 24 h,°C	36.3 ± 0.6	36.8 ± 0.6	< 0.0001
Systemic fibrinolysis, %	18.3	4.4	< 0.0001
Thrombectomy, %	4.4	7.7	0.089
Endovascular treatment, %	4.1	3.4	0.599
Time detection-rtPA, min	172.1 ± 78.3	186.1 ± 91.5	0.079
DWI volume, ml	51.7 ± 98.2	49.3 ± 96.9	0.895
Clinical-DWI mismatch, %	15.1	17.9	0.419
TOAST criteria			0.215
- Atherothrombotic, %	23.1	20.8	
- Cardioembolic, %	36.9	37.5	
Lacunar, %	8.5	6.1	
- Indeterminate, %	30.2	34.1	
- Others, %	1.2	1.5	
**Molecular markers**			
Blood glucose (at admission), mg/dl	142.8 ± 62.7	142.2 ± 54.5	0.815
Glycosylated hemoglobin, %	6.3 ± 1.4	6.1 ± 1.3	0.326
Erythrocyte sedimentation rate, mm	26.4 ± 23.5	33.4 ± 23.8	< 0.0001
LDL cholesterol, mg/dl	106.4 ± 34.4	110.4 ± 39.1	0.129
HDL cholesterol, mg/dl	43.7 ± 17.2	42.2 ± 10.7	0.052
Triglycerides, mg/dl	113.9 ± 54.8	117.5 ± 65.9	0.892
Leukocytes at admission, × 10^3^/ml	9.1 ± 3.2	10.1 ± 3.1	< 0.0001
Fibrinogen at admission, mg/dl	445.0 ± 104.4	465.3 ± 94.6	0.001
C-reactive protein at admission, mg/l	3.4 ± 4.1	6.2 ± 3.6	< 0.0001
IL-6 at admission, pg/ml	27.8 ± 18.6	51.5 ± 15.1	< 0.0001
25-Hydroxy-Vitamin D levels at 24 h, ng/ml	19.2 ± 9.4	5.6 ± 5.8	< 0.0001
Glutamate at admission, μM	267.2 ± 134.7	266.8 ± 99.1	0.124
Glutamate at 24 hours, μM	131.8 ± 80.0	136.7 ± 48.3	0.198
Miocroalbuminuria, mg/24 h	6.5 ± 23.5	4.5 ± 20.1	0.643
NT-proBNP levels at admission, pg/ml	1871.2 ± 2120.9	2012.6 ± 2224.5	0.004
**Outcome**			
Poor outcome, %	48.1	59.1	< 0.0001
NIHSS (at 3 months)	2 [2, 6]	2 [2, 6]	0.225
mRS (at 3 months)	2 [0, 3]	3 [1, 4]	< 0.0001
Early neurological improvement in reperfused patients, %	30.3	27.0	0.215
Early neurological deterioration, %	5.7	3.8	0.107

### Demographic, clinical, and neuroimaging variables

**Fig. 1:**
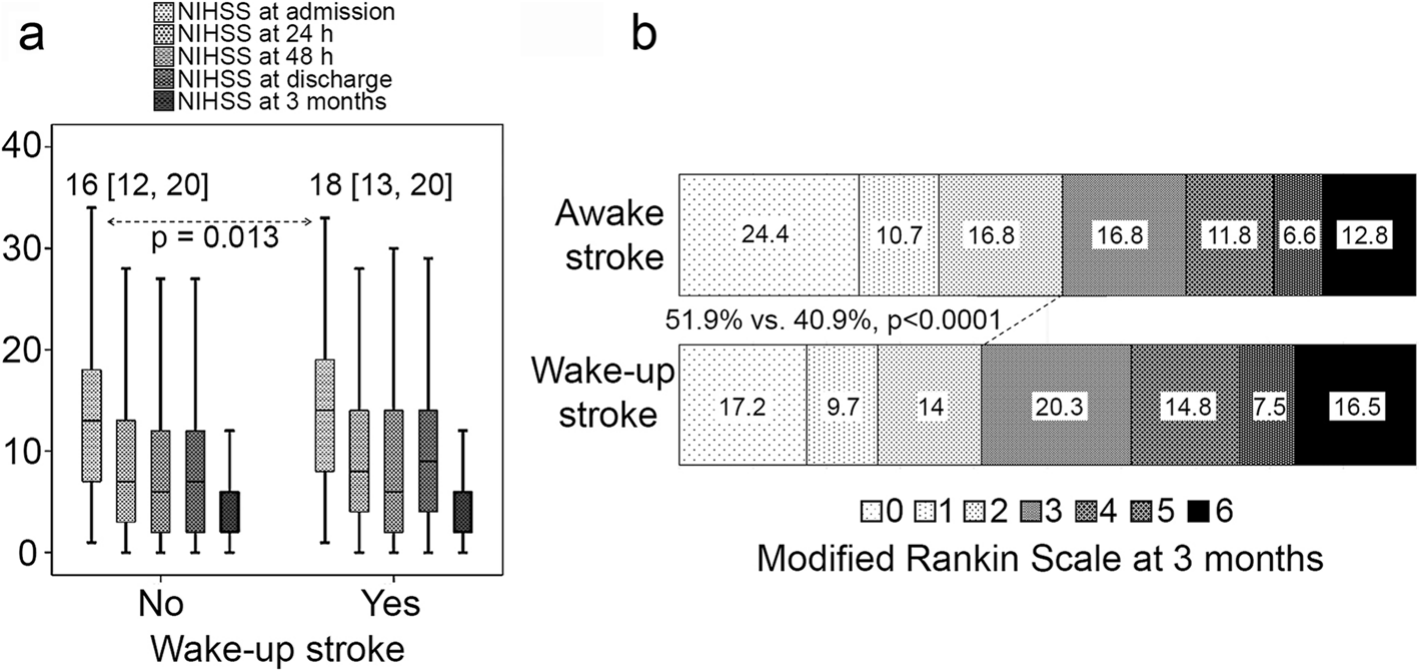
**a** NIHSS evolution of awake and wake-up IS patients. **b** Distribution of the modified Rankin Scale (mRS) scores in IS patients categorized by the stroke onset time

Wake-up stroke patients were younger, showed a higher proportion of atrial fibrillation, hyperlipidemia, or treatment with anticoagulants. No significant differences were found for sex, smoking and alcohol use, as well for other risk factors. According to clinical variables, wake-up IS patients showed a higher proportion of previous poor functional outcome [0 [0, 2] vs. 0 [0, 1], *p* < 0.0001], higher NIHSS at admission [18 [13, 20] vs. 16 [12, 20], *p* = 0.013], at 24 hours [8 [4, 14] vs. 7 [3, 13], *p* = 0.003], and at discharge [9 [3, 14] vs. 7 [2, 12], *p* < 0.0001]. As to early neurological improvement and early neurological deterioration in reperfused patients, there were no significant differences associated to time from stroke onset among patients. Finally, wake-up IS patients showed poor functional outcome at 3 months (59.1% vs. 48.1%, *p* < 0.0001), and higher mRS [3 [1, 4] vs. 2 [0, 3], *p* < 0.0001]. Figure 1a-b details the NIHSS evolution at different time-points between unknown-onset and known-onset IS patients, and the distribution of the mRS scores categorized by the stroke onset time. Patients with wake-up IS have higher neurological deficits at admission, but their clinical course is similar to those patients with awake IS at 3 months. It is important to emphasize that patients with mRs scores of 5/6 and fatal outcome were more common in the wake-up IS group (24.0% vs. 19.4%).

We found differences in the maximum axillary temperature during the first 24 hours (36.3 ± 0.6 °C in awake IS patients vs. 36.8 ± 0.6 °C in wake-up IS patients; *p* < 0.0001), and in the systemic fibrinolysis (intravenous rtPA) (24.3% in awake IS patients vs. 12.1% in wake-up IS patients; *p* < 0.0001). However, no significant differences between groups were found concerning the IS subtype, with a slight superiority of cardioembolic strokes (36.9% vs. 37.5%). In the same line, there was no difference in the time between the detection of symptoms and the administration of rtPA in both groups of patients (186.1 ± 91.5 minutes in wake-up IS group vs. 172.1 ± 78.3 minutes in awake IS group; *p* = 0.079). Furthermore, no difference in DWI volume at admission was found (49.3 ± 96.9 ml in wake-up IS patients vs. 51.7 ± 98.2 ml in awake IS patients; *p* = 0.895), in addition the number of patients with a clinical-DWI mismatch was similar (17.9% vs. 17.1%; *p* = 0.419).

### Association between wake-up IS and different biomarkers

Molecular markers analysis indicated that wake-up IS patients had higher levels of inflammation variables (fibrinogen, leukocytes, C-reactive protein, erythrocyte sedimentation rate, IL-6, and vitamin D), and atrial dysfunction marker (NT-proBNP), highlighting the increased levels of IL-6 at admission (51.5 ± 15.1 pg/ml vs. 27.8 ± 18.6 pg/ml; *p* < 0.0001), as well as the low serum vitamin D levels (5.6 ± 5.8 ng/ml vs. 19.2 ± 9.4 ng/ml; *p* < 0.0001). In contrast, there were no stroke onset time differences related to the excitotoxicity marker glutamate at admission (266.8 ± 99.1 μM in wake-up IS patients vs. 267.2 ± 134.7 μM in awake IS patients; *p* = 0.124), and at 24 hours (136.7 ± 48.3 μM in wake-up IS patients vs. 131.8 ± 80.0 μM in awake IS patients; *p* = 0.198).

**Fig. 2:**
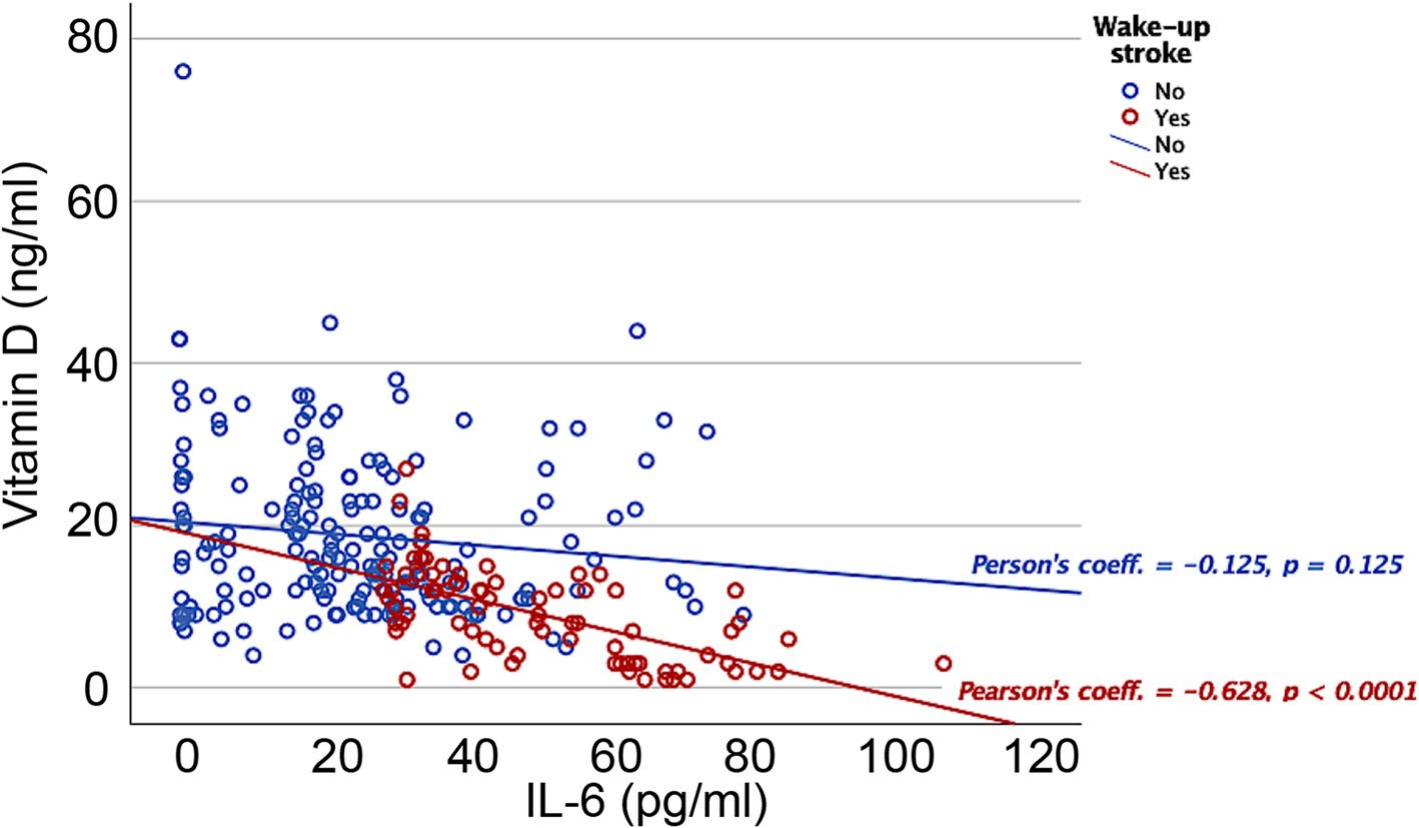
Association between vitamin D and serum levels of IL-6 at admission for wake-up and awake IS patients

From the adjusted logistic regression model detailed in Table 2, Model A, we determined that wake-up IS is independently associated with the following inflammation markers; temperature (OR: 2.15; CI 95%: 1.77–2.61, *p* < 0.0001), fibrinogen (OR: 0.99; CI 95%: 0.99–1.00, *p* = 0.013), C-reactive protein (OR: 1.14; CI 95%: 1.01–1.17, *p* < 0.0001), and vitamin D (OR: 0.75; CI 95%: 0.71–0.79, *p* < 0.0001). However, if we include the serum IL-6 levels in the model, the significance of the previous inflammation markers almost completely disappears, but it is significant for the IL-6 (OR: 1.1; CI 95%: 1.04 1.18, *p* = 0.002) as we can see in Table 2, Model B. Then, high plasma levels of IL-6 are associated with an increased risk of presenting a wake-up IS; the inclusion of IL-6 levels cancels the association of vitamin D levels with stroke upon waking. In Fig. 2**,** an association between vitamin D and serum levels of IL-6 at admission for wake-up and awake IS patients was showed. Low vitamin D levels were related with elevated serum levels of IL-6.

**Table 2:** Logistic regression analysis taking into account clinical factors and biomarkers. Dependent variable: Wake-up IS

**Model A**						
**Independent variables**	**Not adjusted**			**Adjusted**		
	**OR**	**CI 95%**	***p *value**	**OR**	**CI 95%**	***p *value**
Age	1.01	1.00–1.00	0.003	0.99	0.98–1.01	0.879
Previous mRS	1.40	1.20–1.63	< 0.0001	1.25	1.00–1.56	0.049
Hyperlipidemia	1.29	1.05–1.59	0.017	1.19	0.89–1.57	0.226
Atrial fibrillation	1.37	1.08–1.73	0.008	1.06	0.72–1.55	0.783
Anticoagulants	1.74	1.27–2.39	0.001	1.29	0.78–2.13	0.325
Temperature	2.46	2.13–2.84	< 0.0001	2.15	1.77–2.61	< 0.0001
Leukocytes	1.09	1.06–1.12	< 0.0001	1.00	0.96–1.05	0.919
Fibrinogen	1.00	1.00–1.00	0.001	0.99	0.99–1.00	0.013
C-reactive protein	1.13	1.11–1.16	< 0.0001	1.14	1.01–1.17	< 0.0001
Erythrocyte sedimentation rate	1.01	1.00–1.02	< 0.0001	1.00	0.99–1.00	0.688
NIHSS at admission	1.02	1.00–1.03	0.009	0.99	0.98–1.02	0.642
Systemic fibrinolytic treatment	0.43	0.32–0.58	< 0.0001	0.36	0.24–0.54	< 0.0001
NT-proBNP levels	1.00	1.00–1.00	0.005	1.00	1.00–1.00	0.172
25-Hydroxy-Vitamin D at 24 h	0.69	0.66–0.74	< 0.0001	0.75	0.71–0.79	< 0.0001
**Model B**						
**Independent variables**	**Not adjusted**			**Adjusted**		
	**OR**	**CI 95%**	***p *value**	**OR**	**CI 95%**	***p *value**
Age	1.01	1.00–1.00	0.003	0.98	0.96–1.04	0.913
Previous mRS	1.40	1.20–1.63	< 0.0001	1.22	0.99–2.13	0.108
Hyperlipidemia	1.29	1.05–1.59	0.017	1.24	0.68–1.85	0.318
Atrial fibrillation	1.37	1.08–1.73	0.008	1.09	0.70–1.61	0.719
Anticoagulants	1.74	1.27–2.39	0.001	1.27	0.67–2.88	0.403
Temperature	2.46	2.13–2.84	< 0.0001	1.87	1.21–3.15	0.048
Leukocytes	1.09	1.06–1.12	< 0.0001	1.00	0.82–1.13	0.806
Fibrinogen	1.00	1.00–1.00	0.001	1.01	0.86–1.78	0.162
C-reactive protein	1.13	1.11–1.16	< 0.0001	1.13	0.99–1.32	0.089
Erythrocyte sedimentation rate	1.01	1.00–1.02	< 0.0001	0.99	0.72–1.35	0.544
NIHSS at admission	1.02	1.00–1.03	0.009	0.99	0.99–1.06	0.405
Systemic fibrinolytic treatment	0.43	0.32–0.58	< 0.0001	0.67	0.35–0.83	0.018
NT-proBNP levels	1.00	1.00–1.00	0.005	1.00	1.00–1.00	0.172
IL-6 levels at admission	1.12	1.04–1.21	0.003	1.10	1.04–1.18	0.002
25-Hydroxy-Vitamin D at 24 h	0.69	0.66–0.74	< 0.0001	0.85	0.71–1.03	0.089

### Wake-up IS stroke risk and vitamin D levels

**Fig. 3:**
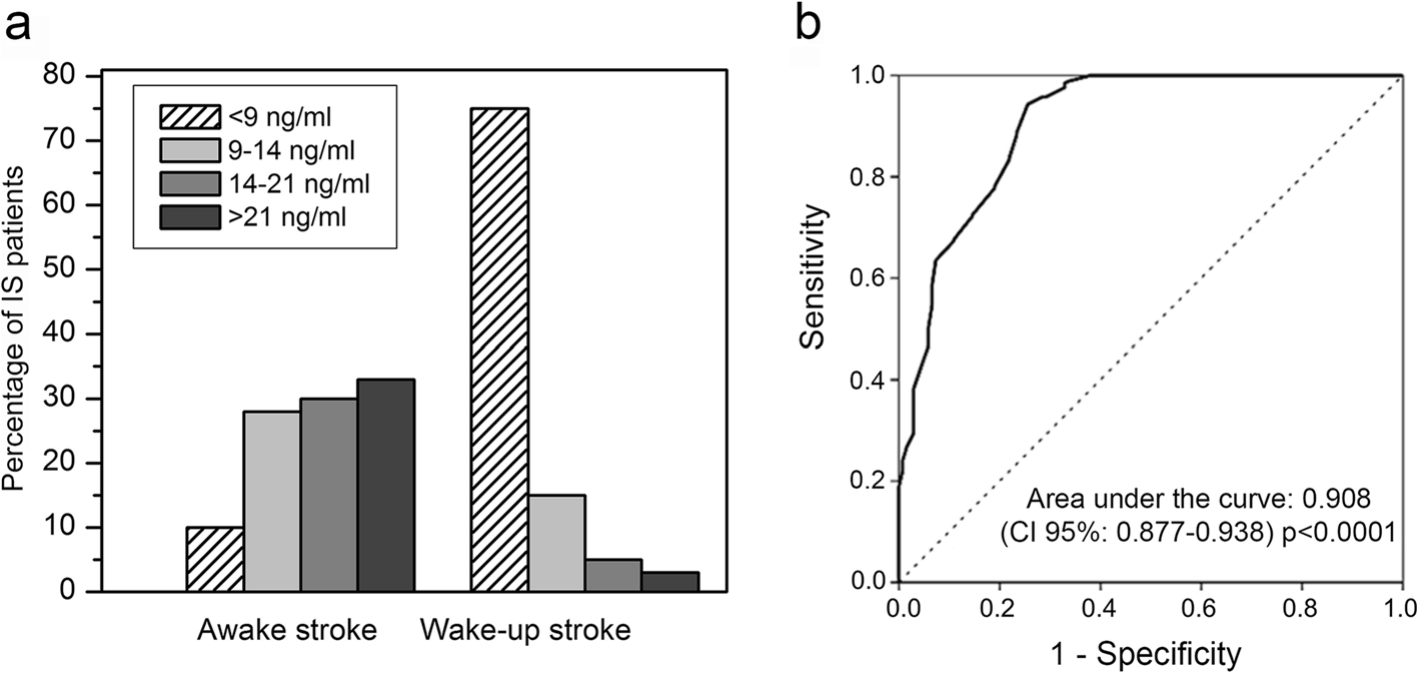
**a** Distribution of serum vitamin D levels quartiles for both IS patient groups. **b** ROC curve analysis to establish the sensitivity and specificity of serum vitamin D levels to predict wake-up IS risk. The cut-off point of vitamin D that optimally predicted a wake-up stroke was 9 ng/ml (area under curve 0.908, sensitivity 88%, specificity 75%, *p* < 0.0001)

We found a significant association between wake-up IS patients and low serum vitamin D levels (9–14 ng/ml, *p* < 0.0001). Figure 3a details the distribution of vitamin D level quartiles for both groups of stroke patients studied. The ROC curve analysis of vitamin D concentrations for wake-up IS showed an area under the curve of 0.908; CI 95%: 0.877–0.938; *p* < 0.0001 (Fig. 3b). For a cut-off point of vitamin D levels ≤9 ng/ml the sensitivity is 88% and the specificity is 75%. On the other hand, vitamin D levels ≤14 ng/mL determine a sensitivity of 64% and a specificity of 93%. In a logistic regression model adjusted only for vitamin D, we found that serum vitamin D concentrations ≤9 ng/ml multiplied by 15 the risk of suffering a wake-up IS (OR: 15.1; CI 95%: 8.6–26.3, *p* < 0.0001).

**Fig. 4:**
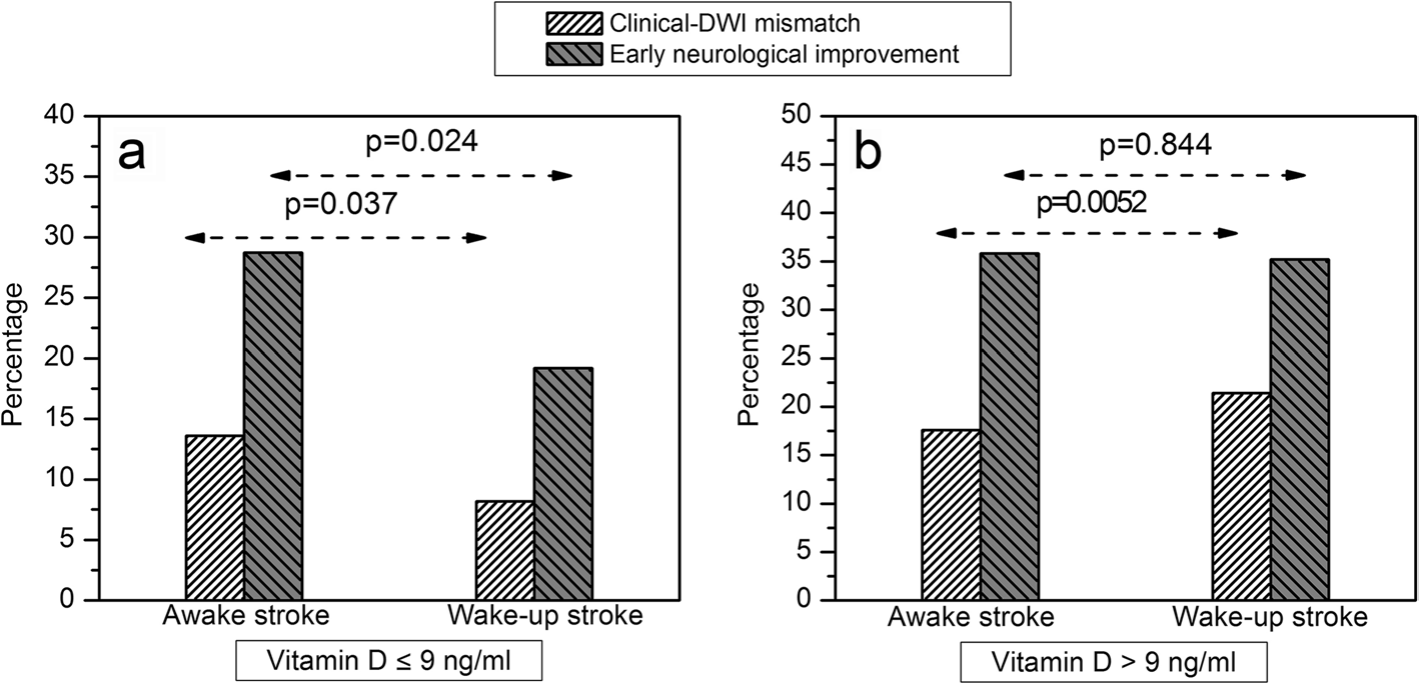
Clinical-DWI mismatch and early neurological improvement relationship patients with IS according to (**a**) vitamin D level < 9 ng/ml, and (**b**) vitamin D level > 9 ng/ml

In turn, vitamin D levels in patients without early neurological improvement were 15.45 ± 9.66 ng/mL, and 24.84 ± 11.77 ng/mL in those who improved early (*p* = 0.032). In a logistic regression model to analyse variables influencing early neurological improvement, vitamin D levels showed an association with an OR 1.16; CI 95%: 1.02–1.39, *p* < 0.0001 in a model adjusted for age, previous mRS, temperature, C-reactive protein, NIHSS on admission and fibrinolytic treatment. However, we found no difference in vitamin D levels between patients with or without clinical-DWI mismatch (no: 18.95 ± 9.66; yes: 17.84 ± 11.77 ng/mL, *p* = 0.394). This result is reflected in Fig. 4, where we detail the correlation between clinical-DWI mismatch and early neurological improvement in both groups of patients with IS according to the following criteria; 9 ng/ml ≤ serum vitamin D levels < 9 ng/ml. As we can see, higher concentrations of this vitamin are clearly associated with a neurological improvement in the patients with IS.

## Discussion

In this study, clinical variables, neuroimaging features and biomarkers associated to inflammation, atrial dysfunction and endothelial or excitotoxicity were evaluated in order to characterize wake-up IS patients. In our series of patients with acute IS (*n* = 4251), who received appropriate treatment according to clinical management guidelines, wake-up episodes occurred in 9.7% of patients. In wake-up IS, 59.1% had a poor functional status at 3 months mainly because they were excluded from acute stroke therapy options. In line with previous studies, we can say that wake-up stroke patients showed an elevated severity at admission [5–7, 36], but their evolution over 3 months was similar to awake IS patients. No difference was found in DWI volume at admission and in clinical DWI mismatch in both group of patients. Findings that support the current trend that MRI or CT scan could be the best methods for extending the time window for wake-up IS patients [17–19].

The results of our study showed that wake-up IS events were independently associated with inflammation markers such as temperature, fibrinogen and C-reactive protein; however, this association was in turn, dependent on low serum vitamin D levels. Thus, the high levels of inflammatory markers in patients with wake-up stroke could be related to low vitamin D levels. To our knowledge, low levels of vitamin D have been associated with increased cardiovascular mortality, cancer incidence [37], autoimmune diseases such as multiple sclerosis [38], poor prognosis of stroke [39]. Clinical studies have demonstrated that a low serum level of vitamin D is associated with higher risk of stroke and negatively impacts recovery and mortality from stroke [24]. Moreover, a recent study has demonstrated an inverse association with IL-6 and high sensitivity C-reactive protein levels, suggesting a potential anti-inflammatory role for vitamin D in stroke individuals [30, 39]. On the other hand, preclinical data suggest that acute administration of vitamin D can limit infarct progression by modulating post-stroke brain inflammation. Supplementation with vitamin D reduced infarct volume by 50%, reduced of pro-inflammatory mediators IL-6, IL-1β, IL-23a, TGF-β and NADPH oxidase-2 in brains of mice, and increased the expression of the T-regulatory cell marker, Forkhead box-P3 (FoxP3) [40]. The impact of vitamin D deficiency in cerebrovascular diseases may adversely affect endothelial cell function and vascular homeostasis through pleiotropic pro-oxidant (endothelial nitric oxide synthase (eNOS), reactive oxygen species (ROS), upregulation of NADPH oxidases (NOXs)) and pro-inflammatory effects (inflammatory cytokines, nuclear factor kappa-light-chain-enhancer of activated B cells (NF-B), matrix metalloproteinases (MMPs)). In turn, these effects related to vitamin D deficiency can enhance tissue sensitivity to oxidative stress and inflammatory events, with consequent increased susceptibility to the onset and severity of stroke events [22].

To our knowledge, this study is the first study about the difference of vitamin D level between wake-up stroke and awake stroke. We have found that serum vitamin D levels may be associated with an increased risk of wake-up IS, in particular serum vitamin D levels ≤9 ng/ml multiply by 15 the risk of suffering an ischemic stroke during sleep. The cardiovascular system is known to be particularly sensitive to vitamin D levels, which can lead to endothelial dysfunction and vascular abnormalities through a variety of mechanisms, including cytokine release, superoxide migration inhibition, and monocyte adhesion and migration [22]. Taking into account that one of the physiological mechanisms involved to wake-up stroke is the endothelial dysfunction, we hypothesized that the endothelial dysfunction could be the “meeting point”, and the endothelium could be the link between risk factors and vascular lesion due vitamin D deficiency. This, however, will require further studies.

On the other hand, when IL-6 was included in the multivariate regression model, the significance of the markers including vitamin D disappeared. The relationship between inflammation and vitamin D levels is well known [34, 35], so vitamin D could be an excellent therapeutic target to reduce inflammation. We can see that high serum levels of IL-6 are associated with an increased risk of waking-up IS and this risk is, to a large extent, independent of low serum vitamin D levels. Considering the clinical view, the diagnosis and monitoring of vitamin D deficiency could prove a preventive action in routine clinical practice to maintain optimal levels by means of adequate supplementation or healthy habits. However, further research is needed in this area because various factors might influence in the origin of deficiency of vitamin D levels, or an individual’s response to a particular dose of vitamin D (obesity, genetic factors, lifestyle, etc.) [22].

This study has the following limitations: First, it is a prospective single center study, although an elevate sample size were included. Second, despite having a very large total patient sample, IS groups were unbalanced. We consider, however, that it would be important to study the two types of stroke independently. Three, only in 61.2% of patients serum glutamate was analyzed, IL-6 in 80.2% of patients and vitamin D in 79.8% of patients. Four, the wake-up group’s pathophysiological link between inflammation and stroke could be due to causes other than vitamin D levels. Atrial fibrillation has recently been associated to increased systemic inflammation, and it was shown to be considerably more common in the wake-up stroke group in the current study (*p* = 0.011). However, clinical and analytical markers of inflammation (temperature, fibrinogen, C-reactive protein, and IL-6) were not found to be higher in wake-up patients with cardioembolic stroke in our data bank. Finally, IL-6, and serum glutamate determinations were not simultaneous. Different researchers performed the determinations, although always blinded to the clinical/neuroimaging data, and supervised by the same senior researcher. In the same lines with the results for clinical and neurological data.

## Conclusion

Wake-up strokes present significant physiological differences from conventional strokes. These differences could be used for reducing their risk of occurrence, facilitating their identification, and improving the care of these patients during their acute phase. The presence of inflammatory biomarkers is the main factor strongly associated with wake-up IS episodes. Serum vitamin D levels ≤9 ng/ml multiply by 15 the risk of suffering an ischemic stroke during sleep. While the benefit of vitamin D supplementation on cerebrovascular outcomes requires deeper study, the diagnosis and monitoring of vitamin D deficiency could be a therapeutic target to reduce wake-up ischemic stroke events. This, however, will require further studies.

## Data Availability

Statistical analysis plan is available on request. Data bank is not available for legal and ethical reasons.

## Abbreviations

CT: Computed Tomography
DWI: Diffusion weighted image
eNOS: Endothelial nitric oxide synthase
ESR: Erythrocyte sedimentation rate
FOxP3: Forkhead box-P3
IL-6: Interleukin 6
IS: Ischemic stroke
OR: Odds ratio
PWI: Perfusion weighted image
MMP: Matrix metalloproteinases
MRI: Magnetic Resonance Imaging
mRS: Modified Rankin Scale
NIHSS: National Institute of Health Stroke Scale
NT-proBNP: N-terminal pro-B-type Natriuretic-Peptide
ROC: Receiver Operating Characteristic
ROS: Reactive oxygen species
rtPA: Intravenous recombinant tissue plasminogen activator
